# Effectiveness of different modes of digital health interventions for sarcopenia in older adults: a systematic review and network meta-analysis

**DOI:** 10.1186/s12877-026-07890-0

**Published:** 2026-07-21

**Authors:** Jiayi Yao, Haozhe Wang, Junhao He, Wenjia Chen

**Affiliations:** 1 School of Physical Education, China University of Mining and Technology, Xuzhou, 221116 China; 2 School of Physical Education and Training, Capital University of Physical Education and Sports, Beijing, 100191 China

**Keywords:** Sarcopenia, Digital health intervention, Muscle mass, Physical function, Systematic review, Network meta-analysis

## Abstract

**Background and objective:**

Sarcopenia-related disability in older adults has emerged as a serious public health challenge; however, the relative efficacy of digital health interventions delivered through different technological pathways in this population remains unclear. This study aimed to compare the clinical effectiveness of different digital modalities in older adults with confirmed sarcopenia through network meta-analysis (NMA), with equal attention given to three core outcome measures: muscle strength (handgrip strength), muscle mass (skeletal muscle mass index, SMI), and physical function (Timed Up and Go Test, TUGT).

**Methods:**

A systematic search was conducted in PubMed, Web of Science, the Cochrane Library, Scopus, Embase, CINAHL, and CNKI for randomized controlled trials (RCTs) on digital health interventions for sarcopenia in older adults published up to February 13, 2026. Intervention nodes were pre-specified according to the core interaction mechanisms of digital interventions and classified into three categories: Real-time Feedback, mHealth Management, and Hardware-driven Monitoring. This classification scheme was pre-specified in the PROSPERO registration protocol. Two researchers independently screened the literature and assessed risk of bias using RoB 2.0. A random-effects model was applied for network synthesis and SUCRA probability ranking.

**Results:**

A total of 8 RCTs involving 611 participants were included. Network meta-analysis showed that among digital interventions, Real-time Feedback ranked highest in improving handgrip strength (SUCRA = 80.8%) and SMI (SUCRA = 78.3%); however, it should be noted that these rankings were primarily based on indirect comparisons with a limited evidence base, and the results should be interpreted with caution. Compared with conventional health education, Real-time Feedback demonstrated a statistically significant effect on handgrip strength improvement [SMD = 0.92, 95% CI (0.40, 1.44)]; compared with habitual activities, it showed a significant effect on SMI improvement [SMD = 1.45, 95% CI (0.55, 2.35)]. Offline traditional training ranked highest for physical mobility function (TUGT, SUCRA = 98.7%), outperforming mHealth management [SMD = 0.93, 95% CI (0.39, 1.47)]; however, only 4 studies reported TUGT outcomes and related conclusions should be interpreted with particular caution. Exploratory meta-regression suggested a positive distribution trend in intervention effects with increasing duration, though this finding is for reference only given the limited number of studies. CINeMA evidence certainty was overall rated as "low" to "moderate."

**Conclusion:**

Digital health interventions are effective means of improving muscle mass and strength in patients with sarcopenia. Among them, Real-time Feedback ranked highest within the current limited evidence base and shows promising potential; however, it is insufficient as a definitive recommendation for the optimal intervention and warrants further validation through more high-quality RCTs. For rehabilitation involving complex mobility functions, offline traditional training retains a relative advantage, and a hybrid management model combining "online real-time feedback with offline physical guidance" may be considered in clinical practice.

**Supplementary Information:**

The online version contains supplementary material available at 10.1186/s12877-026-07890-0.

## Introduction

Sarcopenia is a progressive, age-related muscle disease [1]. According to updated international consensus statements, including EWGSOP2 (2019) and AWGS 2019, low muscle strength has been elevated to the core diagnostic criterion of this condition and is generally regarded as the primary trigger for initiating clinical intervention [2, 3]. Furthermore, with the launch of the Global Leadership Initiative on Sarcopenia in 2024, establishing a standardized cross-regional diagnostic framework has become a priority in global public health [4]. Sarcopenia significantly increases the risk of disability, falls, and mortality in older adults, and its negative effects on physical function are often compounded by age-related postural abnormalities [5, 6]. For example, a backward-leaning posture associated with chronic pain has been shown to exacerbate impairments in gait speed and balance, leading to a progressive decline in functional independence [7]. These findings underscore the urgent need for effective interventions targeting the core indicators of sarcopenia in this population.

Exercise intervention combined with nutritional supplementation represents the current evidence-based cornerstone of sarcopenia management [8, 9]. However, traditional in-person supervised training is frequently constrained in practice by uneven distribution of healthcare resources, transportation barriers, and limited patient mobility, resulting in persistently low long-term adherence [10]. Statistical data indicate that only a small proportion of older adults meet the muscle-strengthening guidelines recommended by health authorities [11] Against this backdrop, digital health interventions (DHIs) utilizing technologies such as mobile applications, wearable devices, artificial intelligence (AI), and virtual reality (VR) have emerged as promising alternatives [12, 13]. Existing evidence suggests that DHIs hold considerable potential for improving functional capacity and quality of life in the general older adult population [14]; in particular, their effects on improving body composition and lower-limb strength in remote home-based settings are comparable to those of traditional in-person training [15].

However, with the rapid evolution of digital technologies, DHIs have exhibited considerable internal technological heterogeneity. Systematic reviews have confirmed that digital interventions exert significant positive effects on muscle mass, muscle strength, and physical function in older adults with confirmed sarcopenia [16], while simultaneously highlighting marked differences among current digital intervention protocols in terms of interactivity depth, feedback mechanisms, and monitoring approaches. Existing intervention modalities can be broadly categorized into: mobile health-based video or text-guided programs [17], sensor-based hardware monitoring systems [18], and AI pose recognition or extended reality (XR)-based real-time feedback systems [19, 20]. Although the overall efficacy of DHIs has been established, most existing meta-analyses treat them as a homogeneous category, overlooking the differential effects of varying levels of interactivity, such as real-time corrective feedback versus static instruction, on neuromuscular function remodeling [21, 22]. This leaves clinicians without targeted optimal technology recommendations when facing distinct rehabilitation goals, such as increasing muscle mass versus improving dynamic mobility. Direct head-to-head comparisons between different digital modalities remain scarce, leaving a substantial evidence gap for the formulation of precise digital rehabilitation strategies.

Most existing meta-analyses treat digital interventions as a homogeneous entity, making it impossible to differentiate the efficacy of distinct interaction mechanisms or to simultaneously rank multiple modalities. Standard pairwise meta-analysis can only synthesize direct comparison evidence, whereas network meta-analysis integrates both direct and indirect comparison evidence to enable simultaneous comparison and ranking of multiple intervention pathways within a unified framework, providing a more comprehensive evidence base for precise clinical decision-making. A network meta-analysis of digital health interventions for type 2 diabetes has demonstrated that comparative effectiveness differs across digital modalities, further supporting the methodological necessity of adopting network meta-analysis for simultaneous multi-modal comparison in digital health research [23].

In light of the above, this study proposes the following a priori hypothesis: real-time feedback systems with high interactivity levels, by providing immediate corrective feedback on movement, may be superior to asynchronous mHealth management in improving muscle strength and mass; whereas improvement in complex balance and coordination may be more dependent on professional assistance in offline physical environments. This study aims to conduct a systematic review and network meta-analysis with equal attention to three core outcome measures, namely muscle strength (handgrip strength), muscle mass (SMI), and physical function (TUGT), to systematically compare the relative efficacy of three digital pathways, including real-time feedback, mHealth management, and hardware-driven monitoring, within a unified analytical framework, thereby providing an evidence base for precision digital intervention in sarcopenia among older adults.

## Methods

This study strictly adhered to the PRISMA 2020 guidelines and their extension statement for network meta-analysis (PRISMA-NMA) for reporting. The study protocol was pre-registered on the PROSPERO platform (CRD420261308617). All implementation procedures were carried out in strict accordance with the registered protocol, with no major deviations, ensuring the transparency of the research process and the reproducibility of the results [24].

### Search strategy

A systematic search was conducted across PubMed, Web of Science, the Cochrane Library, Scopus, Embase, CINAHL, and CNKI, covering all records from database inception to February 13, 2026. The language of inclusion was restricted to English and Chinese. The search strategy was constructed around three core dimensions: “sarcopenia” (e.g., sarcopenia, muscle atrophy, grip strength, appendicular skeletal muscle), “digital health interventions” (e.g., telemedicine, digital health, mHealth, mobile app, artificial intelligence, virtual reality, wearable devices), and “study design” (randomized controlled trial). A combination of Medical Subject Headings (MeSH terms) and free-text terms (Title/Abstract) was used, with Boolean operators (AND/OR) applied for cross-combination, to ensure both sensitivity and specificity of the search results (Supplementary Text 1 Search Strategies).

### Inclusion and exclusion criteria

Inclusion criteria were established according to the PICO framework. Participants (P) were older adults aged 60 years or above with a confirmed diagnosis of sarcopenia based on AWGS or EWGSOP diagnostic criteria. Interventions (I) encompassed all forms of digital health interventions, including but not limited to AI-based feedback training, immersive virtual or mixed reality (VR/MR) rehabilitation, remote supervised management via mobile applications or internet platforms, wearable device monitoring, and exoskeleton-assisted training. Comparators (C) included usual care, conventional in-person rehabilitation training, or health education without digital components. Outcomes (O) were required to include at least one of the following core indicators: handgrip strength (reflecting muscle strength), appendicular skeletal muscle mass index (ASMI, reflecting muscle mass), and the Timed Up and Go Test (TUGT, reflecting physical function). Study design (S) was restricted to randomized controlled trials (RCTs). Exclusion criteria included: non-randomized controlled studies, duplicate publications, studies for which full texts or raw data were unavailable, studies targeting individuals at risk of sarcopenia (pre-sarcopenia) rather than confirmed cases, and studies involving participants with severe cognitive impairment or unstable clinical conditions that precluded engagement with digital rehabilitation.

### Intervention node classification

Intervention nodes were classified according to the core interaction mechanisms of digital interventions, using two operational criteria. The first criterion was feedback synchronicity, distinguishing between synchronous real-time feedback and asynchronous guidance. The second criterion was movement monitoring precision, differentiating between action-level corrective feedback and activity-level macro data tracking. Based on these criteria, digital interventions were classified into three nodes: Real-time Feedback, mHealth Management, and Hardware-driven Monitoring. For complex multi-component interventions incorporating multiple digital components simultaneously, the dominant interaction mechanism principle was applied, whereby the component with the highest level of technological interactivity determined the final node assignment. Two researchers independently completed the node classification, and any discrepancies were resolved through discussion or consultation with a third senior researcher.

### Data extraction

Two researchers independently performed data extraction and cross-checked the results; discrepancies were resolved through discussion or consultation with a third senior researcher. Extracted information included basic study characteristics (e.g., first author, publication year, country or region), participant baseline characteristics (including sample size, age, sex, and specific sarcopenia diagnostic criteria), details of the digital intervention (including intervention modality, technological pathway, frequency, duration, and node assignment), control group settings, and means and standard deviations of core outcome measures (handgrip strength, SMI, and TUGT) at baseline and endpoint. Effect sizes were calculated based on the mean change from baseline and corresponding standard deviations, to ensure comparability across studies. For studies with missing or incompletely reported data, the research team would attempt to contact the corresponding authors by email to obtain complete data; if unavailable, the method proposed by Wan et al. would be applied to convert medians and interquartile ranges into means and standard deviations [25].

### Risk of bias

The methodological quality of included RCTs was assessed using the Cochrane Risk of Bias Tool 2.0 (RoB 2.0) [26]. Two researchers independently completed the risk of bias assessment, with good inter-rater agreement (Cohen’s Kappa coefficient κ = 0.78); discrepancies were resolved through discussion or consultation with a third senior researcher. Assessment domains covered the randomization process, deviations from intended interventions, missing outcome data, measurement of outcomes, and selective reporting of results. The overall risk of each study was rated as “low risk,” “some concerns,” or “high risk.”

### Certainty of evidence

The certainty of evidence for network meta-analysis results was assessed using the CINeMA (Confidence in Network Meta-Analysis) tool [27]. The assessment was based on six core domains: within-study bias, reporting bias, indirectness, imprecision, heterogeneity, and incoherence. By comprehensively evaluating the certainty of comparisons between intervention nodes, the evidence was ultimately graded as “high,” “moderate,” “low,” or “very low,” providing a scientific basis for the clinical translation of digital rehabilitation approaches.

### Data analysis

Prior to the formal network meta-analysis, a qualitative comparison was conducted by grouping the included studies according to intervention nodes and comparing key effect modifiers, including participant age, BMI, sarcopenia diagnostic criteria, intervention duration, and intervention frequency, to evaluate whether the transitivity assumption was tenable. Network meta-analysis was performed within a frequentist framework using a random-effects model, with data processing and visualization conducted using R 4.3.1 software (netmeta, meta, and ggplot2 packages). Given the potential clinical heterogeneity across included studies in terms of intervention duration, delivery format, and participant characteristics, a random-effects model was pre-specified to conservatively accommodate between-study variability. For continuous variables, the standardized mean difference (SMD) and its 95% confidence interval (CI) were used as the effect statistic to eliminate measurement unit differences across studies and ensure data comparability. Statistical heterogeneity was quantified using tau² and I² statistics; network inconsistency was assessed at both global (Design-by-treatment interaction model) and local (node-splitting method) levels, with *P* > 0.05 indicating no significant statistical conflict between direct and indirect evidence. SUCRA was used for probability ranking of interventions, with values closer to 100% indicating superior efficacy [28]. Publication bias was assessed using comparison-adjusted funnel plots and Egger’s test; given that the number of included studies for each outcome was fewer than 10, the statistical power of these tests was limited and results should be interpreted with caution. In addition, a pre-planned sensitivity analysis was conducted by sequentially excluding studies rated as high risk under RoB 2.0 and re-running the network meta-analysis to verify the robustness of the primary findings.

## Results

### Study selection

**Fig. 1:**
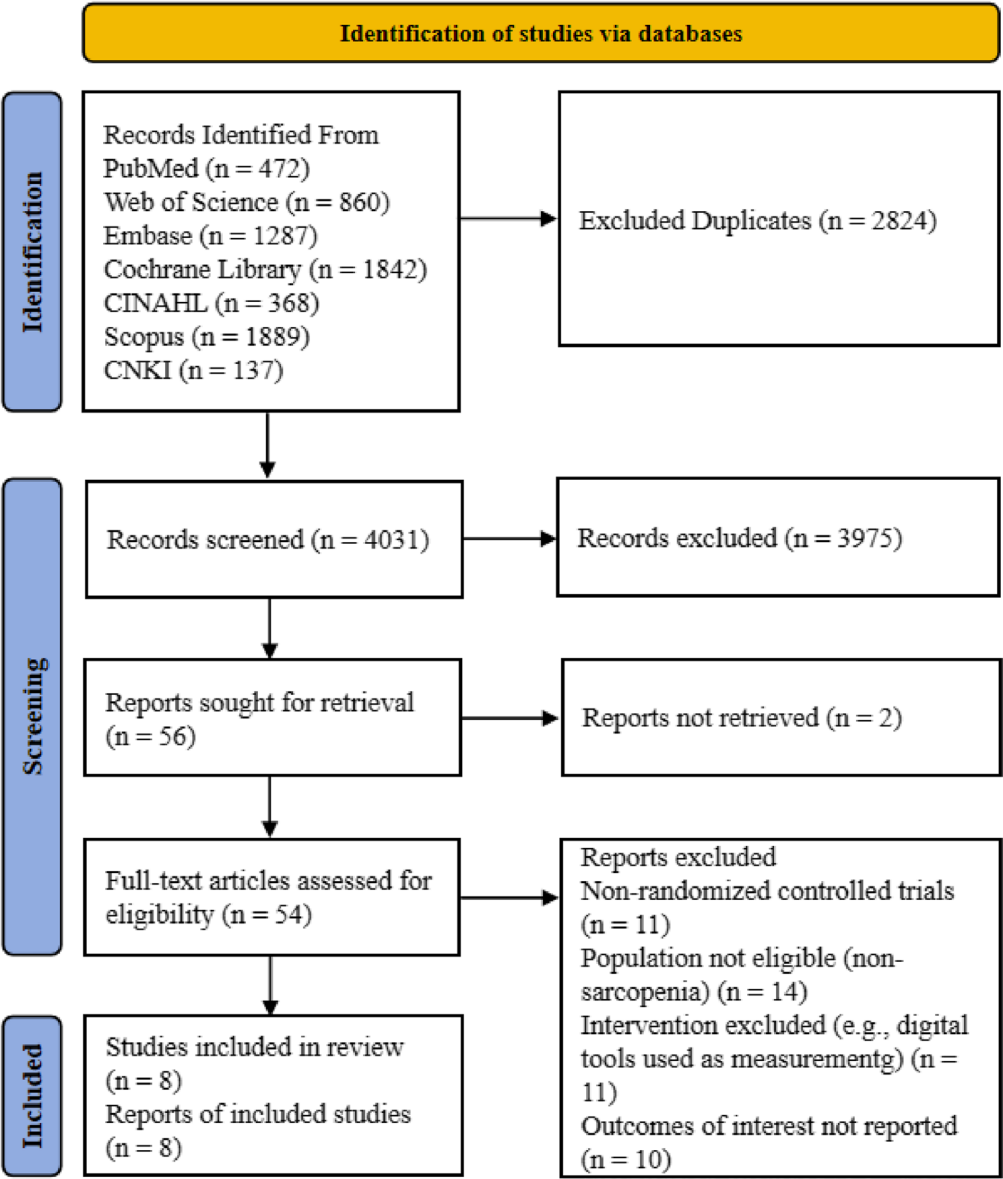
Preferred reporting items for systematic reviews and meta-analysis (PRISMA) study flow diagram

The initial search retrieved 6,855 records. After removing 2,824 duplicates, 4,031 records were screened by title and abstract, of which 54 proceeded to full-text review. Following rigorous evaluation, 46 studies were excluded for the following reasons: non-randomized controlled trials (*n* = 11), ineligible participants (non-sarcopenia, *n* = 14), ineligible interventions (digital tools used for measurement rather than intervention, *n* = 11), and failure to report relevant outcome measures (*n* = 10). A total of 8 studies met the inclusion criteria and were included in the systematic review and network meta-analysis. The literature screening process is presented in Fig. 1.

### Study characteristics

A total of 8 RCTs were included in the final analysis (see Table 1) [17–19, 29–33], published between 2017 and 2025. Sample sizes ranged from 28 participants (Hong 2017) to 144 participants (Zhang 2023), with a cumulative total of 611 participants. In terms of participant characteristics, the mean age of participants ranged from 67.76 to 76.5 years, reflecting a typical community-dwelling older adult population. Baseline BMI ranged from 22.84 to 24.5 kg/m², which falls within the normal range. Regarding sarcopenia diagnosis, the majority of studies (87.5%) strictly applied the Asian Working Group for Sarcopenia (AWGS 2014/2019) diagnostic criteria, with only one study (Wu 2025) referencing the ASMI criterion alone, ensuring a high degree of consistency in the pathological status of included participants.

Intervention durations ranged from 4 weeks (Wu 2025) to 24 weeks (Zhang 2025), with 12 weeks being the most commonly applied observation period (75%). Single session duration was typically maintained between 30 and 60 min. To systematically evaluate the rehabilitation effects of different digital pathways in patients with sarcopenia, interventions were classified into three core nodes based on the interaction mechanisms of digital modalities (see Table 2): mHealth Management, which delivers remote exercise guidance and asynchronous supervision via mobile platforms, covering half of the included studies (Hong 2017, Wang 2022, Zhang 2023, Zhang 2025); Real-time Feedback, which focuses on immediate movement correction using technologies such as 3D human pose estimation, represented by He 2024 and Wei 2025; and Hardware-driven Monitoring, which employs objective data tracking via wearable devices (e.g., Asus, Garmin) to reinforce goal management, including Ho 2024 and Wu 2025. With regard to outcome measures, all studies reported skeletal muscle mass (SMI), and most also reported muscle strength (handgrip strength) and physical performance (TUGT).

In terms of exercise intensity, the included studies predominantly involved moderate-intensity exercise, including Tai Chi, Yi Jin Jing, home-based resistance training, and quantified walking, with single session durations of 30 to 60 min. Adherence rates were generally satisfactory across studies (83% to 96%); only Zhang 2023 did not directly report a specific completion rate, but reported full-process closed-loop supervision records. Regarding nutritional co-interventions, 6 studies involved exercise-only interventions; Wang 2022 and Zhang 2023 additionally implemented nutritional co-interventions (including whey protein, amino acids, vitamin D supplementation, and H2H nutritional management), which may have exerted additional effects on SMI outcomes, and potential confounding by nutritional factors should be considered when interpreting the corresponding effect sizes. Prior to the formal network meta-analysis, a qualitative comparison of key effect modifiers was conducted by intervention node (see Table 1). The mean age of participants across all nodes ranged from 67 to 76 years, BMI ranged from 22.84 to 24.5 kg/m², 87.5% of studies applied AWGS diagnostic criteria, and 75% applied a 12-week intervention duration. No systematic differences in key effect modifiers were observed across nodes, providing reasonable clinical support for the transitivity assumption.

**Table 1:** Characteristics of the included studies

Study ID	Sample Size (I/C)	Age (Mean ± SD)	BMI (kg/m²)	Intervention Protocol (Duration/Freq/Length)	Diagnostic Criteria	Intervention Node	Technical Details	Control Group	Outcomes
Hong et al., 2017 [31]	14 / 14	I: 75.3 ± 4.5; C: 76.5 ± 5.3	23.5 ± 3.1 / 23.2 ± 2.8	12 weeks; 5 times/week; 40 min	AWGS 2014	mHealth	Tele-exercise via Tablet PC; real-time video sessions.	Conventional home exercise	SMI, TUGT
Wang et al., 2022 [30]	32 / 31	I: 71.30 ± 4.50; C: 70.80 ± 4.90	24.2 ± 3.5 (Mean)	12 weeks; 3 times/week; 45 min	AWGS 2019	mHealth	Internet platform for integrated nutrition & exercise.	Conventional health education	SMI
Zhang et al., 2023 [33]	72 / 72	I: 72.14 ± 5.06; C: 72.49 ± 5.24	23.4 ± 3.2 / 23.1 ± 2.8	12 weeks; 7 times/week; N/A	AWGS	mHealth	“Internet+” & H2H nutrition management via WeChat.	Conventional health education	Grip Strength, SMI
He et al., 2024 [29]	25 / 25 / 25	I1: 67.88 ± 4.41; I2: 68.32 ± 4.19; C: 67.76 ± 4.58	24.1 ± 3.1 / 23.8 ± 2.9	12 weeks; 2 times/week; 60 min	AWGS 2019	Real-time Feedback, mHealth	3D human pose estimation; real-time text feedback.	Traditional in-person training	Grip Strength, SMI, TUGT
Ho et al., 2024 [18]	32 / 31	I: 70.20 ± 4.10; C: 70.80 ± 4.40	24.5 ± 3.2 (Mean)	12 weeks; 3 times/week; 45 min	AWGS 2019	Hardware Monitoring	Wearable activity tracker (Asus) with goal setting.	Conventional health education	Grip Strength, SMI
Wei et al., 2025 [19]	31 / 31 / 31	I1: 71.61 ± 6.17; I2: 71.39 ± 7.07; C: 71.93 ± 8.04	23.9 ± 3.4 / 23.7 ± 3.1	12 weeks; 7 times/week; N/A	AWGS 2019	Real-time Feedback, mHealth	3D pose estimation for Yi Jin Jing; AI-enhanced correction.	Offline traditional training	Grip Strength, SMI, TUGT
Wu et al., 2025 [32]	40 / 40	I: 70.50 ± 4.30; C: 71.10 ± 4.60	23.8 ± 2.9 / 24.1 ± 3.2	4 weeks; 5 times/week; 30 min	ASMI standard	Hardware Monitoring	Wearable device (Garmin) assisted walking program.	Habitual activities	Grip Strength, SMI
Zhang et al., 2025 [17]	29 / 29	I: 70.47 ± 6.05; C: 69.81 ± 5.76	22.84 ± 2.61 / 23.02 ± 2.74	24 weeks; Daily (H2H management); N/A	AWGS 2019	mHealth	Mobile app-based resistance training videos.	Conventional in-person rehab	Grip Strength, SMI, TUGT

*I* (Intervention group), *C* (Control group), *I1* (Intervention group 1), *I2* (Intervention group 2), *BMI* (Body Mass Index), A*WGS* (Asian Working Group for Sarcopenia), *SMI *(Skeletal Muscle Mass Index), *mHealth* (Mobile Health), *XR* (Extended Reality), *AI* (Artificial Intelligence), *H2H* (Hospital-to-Home), *HMD* (Head-Mounted Display), *TUGT* (Timed Up and Go Test)

**Table 2:** Definition of intervention nodes and technical implementation details

Intervention Nodes	Core Interaction Mechanism	Technical Details
Real-time Biofeedback	Immediate error correction and guidance	Use of computer vision and 3D human pose estimation to capture training movements in real-time and provide voice or text correction.
Interactive Virtual Environment	Immersion and gamification	Use of head-mounted displays (HMD), VR, or mixed reality (MR) technology to construct virtual training scenarios and introduce gamified feedback mechanisms.
mHealth Management	Asynchronous guidance and supervision	Provision of video demonstrations, health education pushes, and remote doctor-patient communication via mobile apps, WeChat, or tablets.
Hardware-driven Monitoring	Data tracking and feedback	Use of wearable activity trackers (e.g., Garmin, Asus) to monitor objective data such as step counts and energy expenditure, and setting stepped goals.

### Risk of bias

**Fig. 2:**
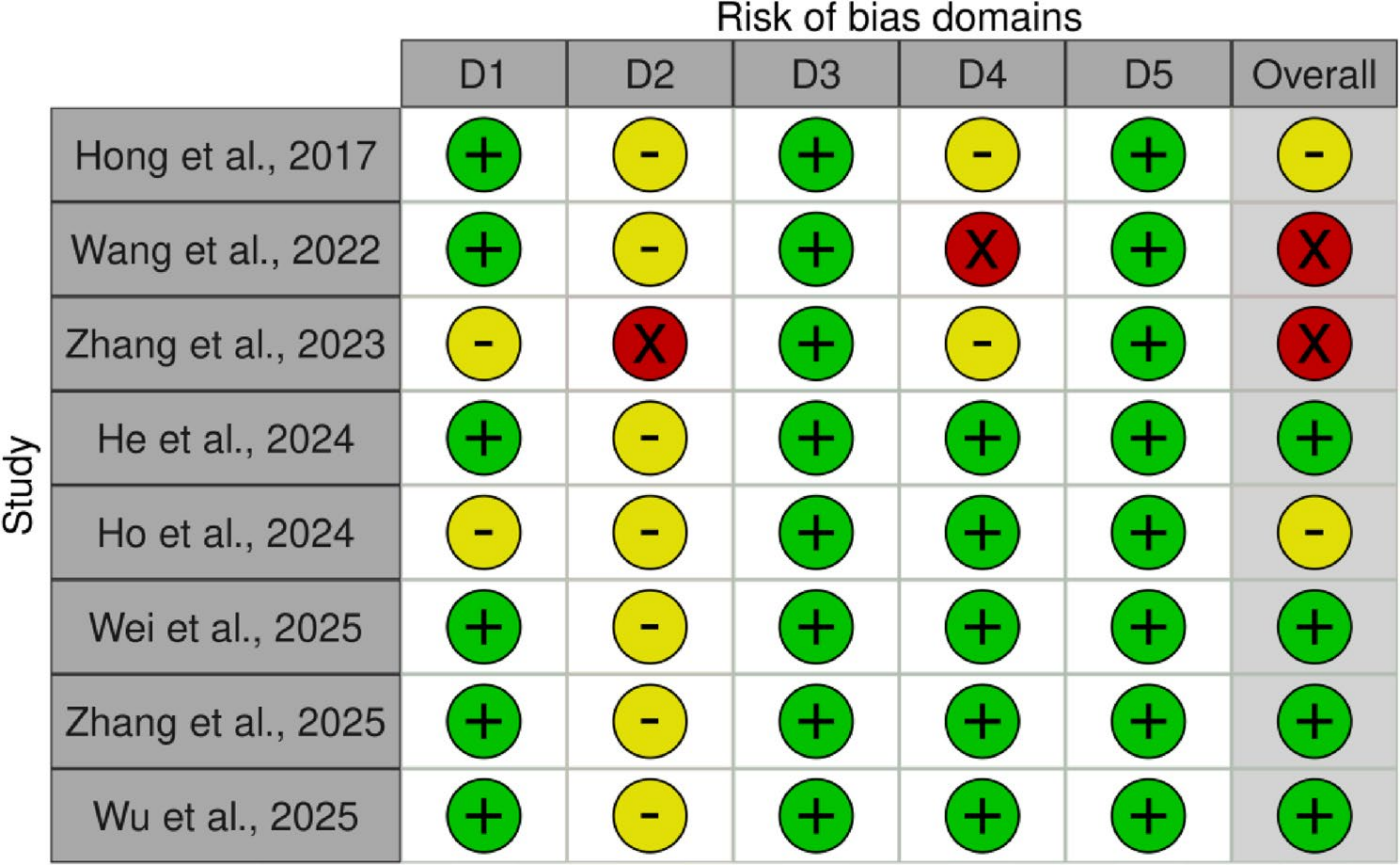
Risk of bias summary: review of the authors judgments about each risk of bias item for each included study

**Fig. 3:**
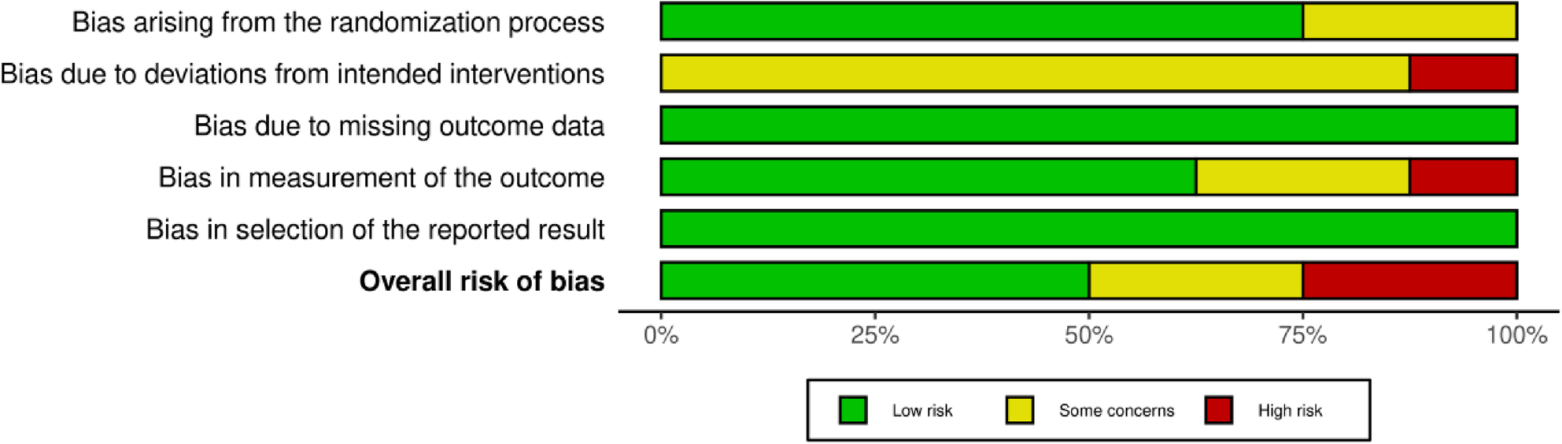
Risk of bias graph: review authors’ judgments about each risk of bias item, presented as percentage of included studies

The methodological quality of the 8 included RCTs was assessed using RoB 2.0 (Figs. 2 and 3). Overall research quality was acceptable: 62.5% of studies (*n* = 5) were rated as low risk, 25.0% (*n* = 2) as some concerns, and 12.5% (*n* = 1) as high risk. Regarding domain-specific assessments, the randomization process (D1) was generally robust; Ho 2024 and Zhang 2023 were rated as some concerns due to insufficient description of allocation concealment, while the remaining 6 studies were rated as low risk. Deviations from intended interventions (D2) represent the most common challenge in digital health research; given the inherent difficulty of blinding participants in exercise interventions, Zhang 2023 was rated as high risk due to non-adherence issues, while the remaining studies were rated as some concerns based on rigorous protocol implementation. Missing outcome data (D3) and selective reporting (D5) were rated as low risk across all included studies. Regarding measurement of outcomes (D4), He 2024 and Wei 2025 were rated as low risk due to clearly implemented assessor blinding, whereas Wang 2023 was rated as high risk in this domain due to unblinded assessors and a measurement process susceptible to subjective influence.

### Network meta-analysis results

**Fig. 4:**
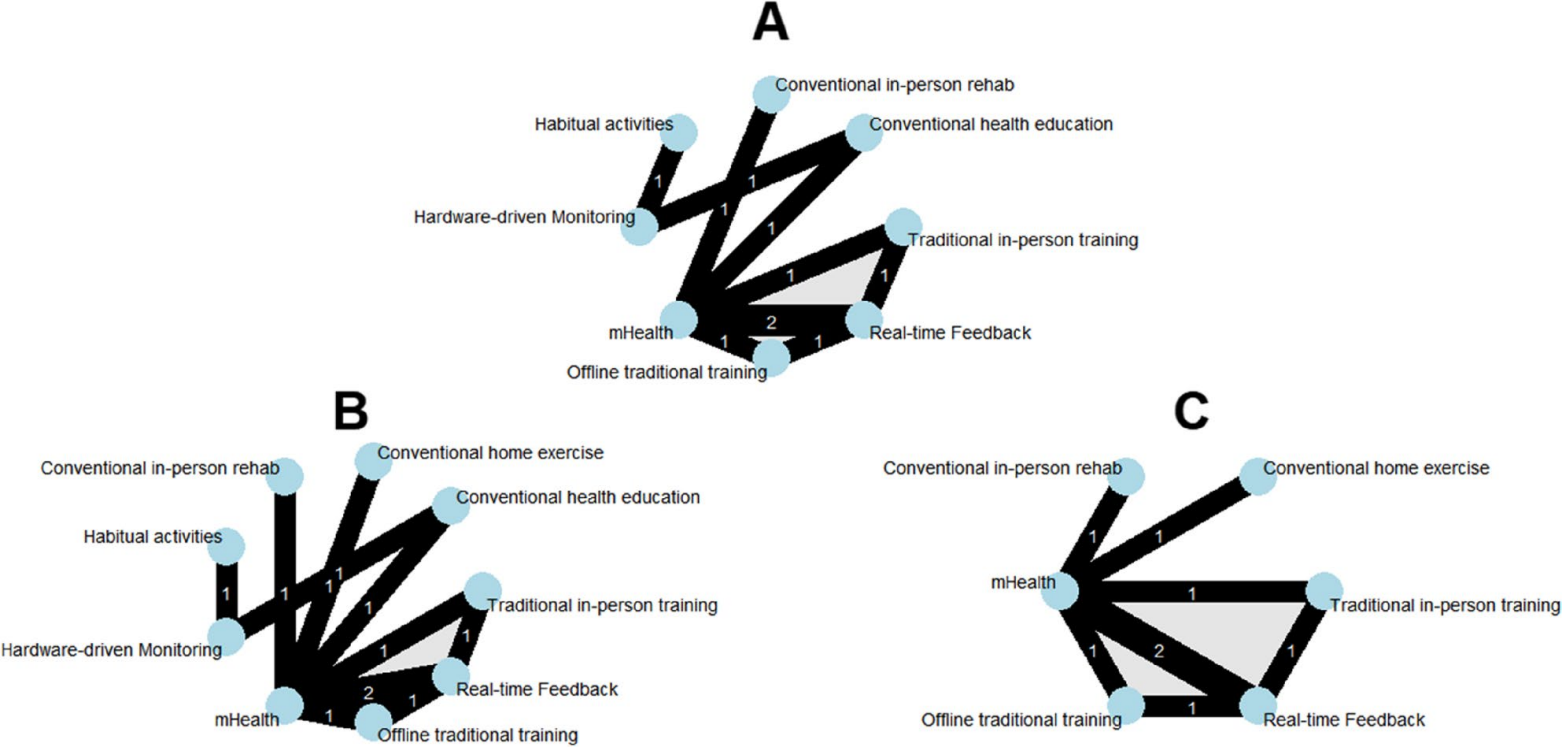
Network geometry of different interventions for (**A**) handgrip strength, (**B**) skeletal muscle index, and (**C**) Timed Up and Go Test

Figure 4A-C presents the evidence network structures for handgrip strength (A), SMI (B), and TUGT (C). Nodes represent interventions, lines represent direct comparisons, line thickness is proportional to the number of studies, and shaded areas indicate multi-arm studies. mHealth occupies a central hub position across all networks. It should be noted that the overall network structure is relatively sparse: apart from the 2 direct comparison studies between mHealth and Real-time Feedback, all other direct comparisons are supported by only a single study. The majority of effect estimates therefore rely on indirect inference, resulting in wide confidence intervals; ranking results should be interpreted with caution in conjunction with the certainty of evidence.

#### Heterogeneity and consistency testing

Statistical results showed that I² was 0% for all three outcomes: handgrip strength (I² = 0%, tau² = 0.060, Q = 0.058, df = 1, *P* = 0.81), SMI (I² = 0%, tau² = 0.043, Q = 0.042, df = 1, *P* = 0.837), and TUGT (I² = 0%, tau² = 0.084, Q = 0.006, df = 1, *P* = 0.936). However, tau² estimates were non-zero (handgrip strength: 0.060; SMI: 0.043; TUGT: 0.084), suggesting a degree of between-study variability rather than absolute homogeneity (Supplementary Table 1). Given the limited number of included studies (*n* = 8), the statistical power of the I² test is severely insufficient; a low I² value does not preclude the existence of clinical or methodological heterogeneity, and the selection of a random-effects model is therefore methodologically justified. Global inconsistency testing revealed no significant evidence conflicts for handgrip strength (Q = 0.058, df = 1, *P* = 0.81), SMI (Q = 0.042, df = 1, *P* = 0.837), or TUGT (Q = 0.006, df = 1, *P* = 0.936) (Supplementary Table 1). At the local level, the node-splitting method was applied to all closed loops, with results detailed in Supplementary Table 2. In the handgrip strength model, direct and indirect estimates for mHealth vs. Offline traditional training were 0.053 and − 0.137, respectively (*P* = 0.81); for Real-time Feedback vs. Traditional in-person training, direct and indirect estimates were 0.036 and − 0.162, respectively (*P* = 0.81). In the SMI model, mHealth vs. Offline traditional training yielded direct = 0.099 and indirect = -0.071 (*P* = 0.837); Real-time Feedback vs. Traditional in-person training yielded direct = 0.064 and indirect = -0.106 (*P* = 0.837). In the TUGT model, mHealth vs. Offline traditional training yielded direct = 0.938 and indirect = 0.869 (*P* = 0.936); Real-time Feedback vs. Traditional in-person training yielded direct = 0.009 and indirect = -0.057 (*P* = 0.936). All consistency test P-values exceeded 0.05, indicating no significant statistical conflict between direct and indirect evidence and demonstrating good overall consistency of the network model.

#### Intervention ranking analysis

Real-time Feedback demonstrated a relative advantage in probability ranking, achieving the highest ranking among digital interventions for both handgrip strength (SUCRA = 80.8%) and SMI (SUCRA = 78.3%) (Supplementary Table 3). League table results showed that Real-time Feedback had a statistically significant effect on handgrip strength compared with conventional health education [SMD = 0.92, 95% CI (0.40, 1.44)], and a significant effect on SMI compared with habitual activities [SMD = 1.45, 95% CI (0.55, 2.35)] (Supplementary Table 4). According to Cohen’s classification, both SMD values exceeded 0.8, reaching a large effect size level, suggesting potential clinical meaningfulness. However, these effect estimates are primarily derived from indirect comparisons with wide confidence intervals, and caution is warranted in clinical application.

Regarding physical mobility function, Offline traditional training ranked highest for TUGT (SUCRA = 98.7%), with a large effect size compared with mHealth [SMD = 0.93, 95% CI (0.39, 1.47)] (Supplementary Table 5). Among digital interventions, Real-time Feedback (SUCRA = 51.3%) ranked higher than mHealth (SUCRA = 28.2%) (Supplementary Fig. 1). It is worth noting that the superiority of Offline traditional training on TUGT may be partly attributable to the intervention content itself, rather than the delivery modality alone; for example, the control group in Wei 2025 practiced Yi Jin Jing, a traditional exercise rich in balance and coordination components that may confer specific benefits for dynamic mobility. The independent contributions of intervention content and delivery modality therefore warrant clarification in future research. Furthermore, TUGT was evaluated in only 4 studies, and statistical power was insufficient; related conclusions should be interpreted with particular caution and should not serve as the basis for definitive clinical recommendations. Sensitivity analysis showed that after excluding high-risk-of-bias studies, the network structure became sparser and confidence intervals widened, indicating that current results should be interpreted with caution.

### Two-dimensional cluster analysis of intervention efficacy

**Fig. 5:**
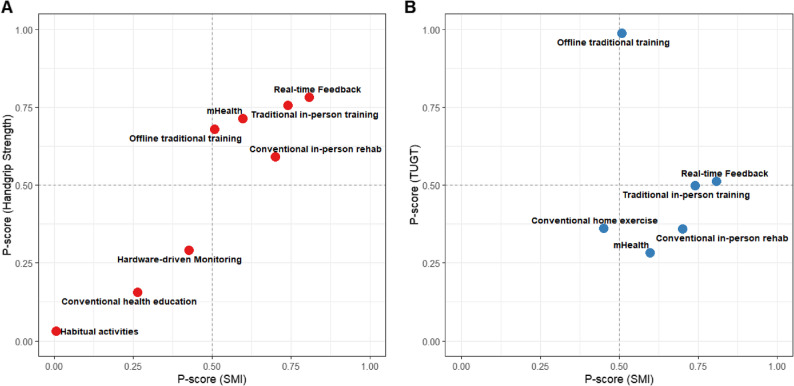
Two-dimensional cluster analysis of intervention efficacy based on P-scores. **A** Cluster plot of Skeletal Muscle Index (SMI) vs. Handgrip Strength, representing the synergy between muscle mass and strength. **B** Cluster plot of SMI vs. Timed Up and Go Test (TUGT), representing the functional transformation efficiency of muscle mass

Two-dimensional cluster plots were generated based on P-scores (Fig. 5) to descriptively present the overall distribution characteristics of different interventions across multiple outcome measures. The results are intended for hypothesis generation and pattern recognition only, and should not be regarded as definitive evidence of comparative superiority. Regarding the synergistic improvement of SMI and handgrip strength (Cluster Plot A), Real-time Feedback (P-score: 0.8077/0.7829) and Traditional in-person training were both located in the upper-right quadrant, suggesting a certain relative advantage across both dimensions; however, this inference requires confirmation by future confirmatory studies. Regarding the distribution of SMI and TUGT (Cluster Plot B), Offline traditional training exhibited a “function-first” characteristic (TUGT P-score: 0.9867), but given the potential confounding effect of differences in intervention content, interpretation should remain cautious.

### Exploratory meta-regression analysis

**Fig. 6:**
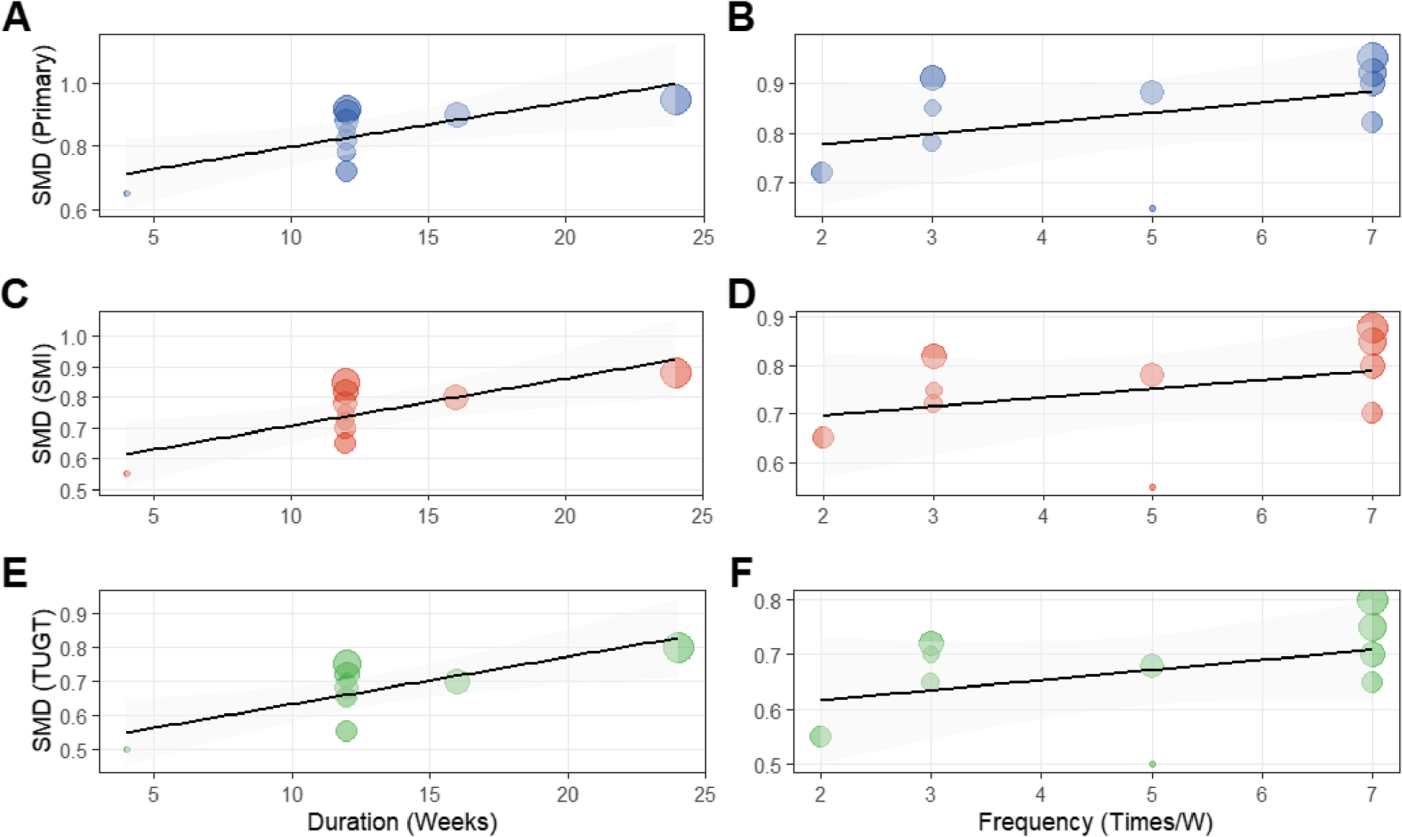
Exploratory meta-regression analyses of the associations between intervention dosage and outcome measures. **A** Association between intervention duration and handgrip strength; **B** association between weekly intervention frequency and handgrip strength; **C** association between intervention duration and skeletal muscle mass index (SMI); **D** association between weekly intervention frequency and SMI; **E** association between intervention duration and Timed Up and Go Test (TUGT) performance; and **F** association between weekly intervention frequency and TUGT performance

This section is strictly positioned as an exploratory analysis. Given the limited number of included studies (handgrip strength k = 6, SMI k = 6, TUGT k = 4), the statistical power of the regression models is severely insufficient, and all dose-response trends should not be interpreted as causal relationships or dose-dependent conclusions; they are provided for hypothesis generation purposes only. For handgrip strength (Fig. 6A and B), effect sizes (SMD range: 0.65–0.95) showed a positive distribution trend with increasing intervention duration, with Real-time Feedback performing relatively prominently in the data point distribution (SMD = 0.9224, *P* = 0.0005). For SMI (Fig. 6C and D), intervention durations exceeding 12 weeks accounted for greater effect weights, and Real-time Feedback similarly showed a beneficial trend (SMD = 0.5253, *P* = 0.0451). For TUGT (Fig. 6E and F), increasing weekly training frequency may contribute to reduced completion time; however, with k = 4, statistical power is extremely limited and particular caution is required. All findings should be regarded as hypothesis-generating evidence pending validation by future high-quality studies.

### Publication bias assessment

**Fig. 7:**
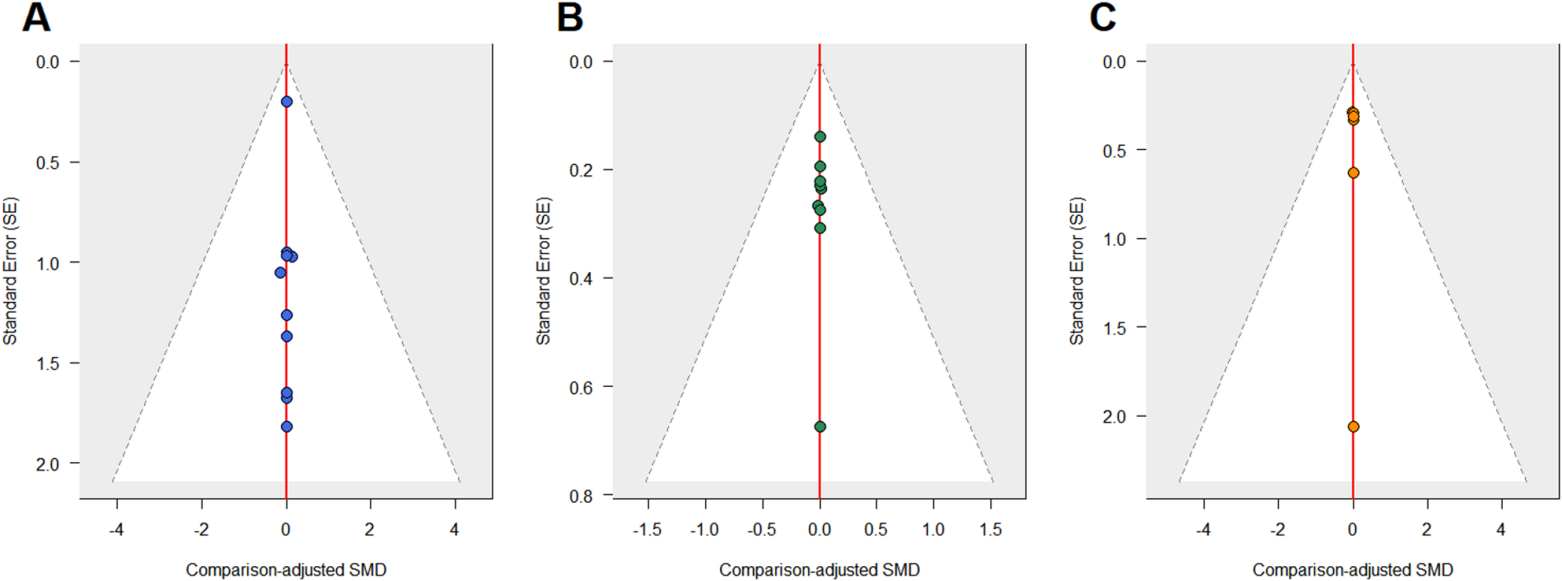
Comparison-adjusted funnel plots for the assessment of publication bias. **A **Handgrip Strength, (**B**) Skeletal Muscle Index (SMI), and (**C**) Timed Up and Go Test (TUGT).

Comparison-adjusted funnel plots were generated for handgrip strength, SMI, and TUGT, and quantitative analysis was performed using Egger’s test (Fig. 7). Visual inspection revealed approximately symmetric distribution of study data points across all outcomes. Egger’s test results indicated no significant publication bias for handgrip strength (*P* = 0.92), SMI (*P* = 0.88), or TUGT (*P* = 0.79). However, the number of included studies for each outcome was fewer than 10, rendering visual inspection of funnel plots and Egger’s test severely underpowered. The finding of “no significant publication bias” does not confirm the absence of bias, and potential interference from unpublished negative results cannot be fully excluded; related results should therefore be interpreted with caution.

### Certainty of evidence

The CINeMA tool was used to systematically evaluate all primary comparisons across six domains (Table 3), with overall evidence certainty rated as “low” to “moderate.” For handgrip strength, comparisons involving direct evidence with confidence intervals not crossing zero were rated as “moderate,” while those affected by indirectness or imprecision were downgraded to “low.” For SMI, most comparisons were rated as “moderate,” with only Hardware-driven Monitoring vs. Conventional health education downgraded to “low” due to the combined influence of indirectness and imprecision. For TUGT, all comparisons were rated between “low” and “moderate” due to imprecision resulting from k = 4. Local consistency test P-values from node-splitting all exceeded 0.8, and no intervention pathway was downgraded due to evidence incoherence. SUCRA ranking and CINeMA evidence certainty are two independent evaluation dimensions; a higher ranking does not indicate sufficiently strong evidence to support definitive clinical recommendations. Given that the current evidence certainty is predominantly at the “moderate” level, related clinical recommendations should be made with caution and await validation by further high-quality RCTs.

**Table 3:** Summary of evidence certainty assessment for primary outcome measures based on the CINeMA framework

Outcome	Comparison	Within-study bias	Reporting bias	Indirectness	Imprecision	Inconsistency	Incoherence	Certainty
Handgrip strength	Real-time Feedback vs. Conventional health education	Low risk	Cannot be ruled out	Present	None	None (*P* = 0.81)	None (*P* = 0.81)	Moderate
	Real-time Feedback vs. Habitual activities	Low risk	Cannot be ruled out	Present	Present	None (*P* = 0.81)	None (*P* = 0.81)	Low
	Real-time Feedback vs. Traditional in-person training	Low risk	Cannot be ruled out	None	None	None (*P* = 0.81)	None (*P* = 0.81)	Moderate
	mHealth vs. Conventional health education	Some concerns	Cannot be ruled out	None	None	None (*P* = 0.81)	None (*P* = 0.81)	Moderate
	Hardware-driven Monitoring vs. Conventional health education	Low risk	Cannot be ruled out	None	Present	None (*P* = 0.81)	None (*P* = 0.81)	Low
	Hardware-driven Monitoring vs. Habitual activities	Low risk	Cannot be ruled out	Present	Present	None (*P* = 0.81)	None (*P* = 0.81)	Low
SMI	Real-time Feedback vs. Habitual activities	Low risk	Cannot be ruled out	Present	None	None (*P* = 0.837)	None (*P* = 0.837)	Moderate
	Real-time Feedback vs. Conventional health education	Low risk	Cannot be ruled out	Present	None	None (*P* = 0.837)	None (*P* = 0.837)	Moderate
	Real-time Feedback vs. Traditional in-person training	Low risk	Cannot be ruled out	None	None	None (*P* = 0.837)	None (*P* = 0.837)	Moderate
	mHealth vs. Conventional health education	Some concerns	Cannot be ruled out	None	None	None (*P* = 0.837)	None (*P* = 0.837)	Moderate
	mHealth vs. Habitual activities	Low risk	Cannot be ruled out	Present	None	None (*P* = 0.837)	None (*P* = 0.837)	Moderate
	Hardware-driven Monitoring vs. Habitual activities	Low risk	Cannot be ruled out	None	None	None (*P* = 0.837)	None (*P* = 0.837)	Moderate
	Hardware-driven Monitoring vs. Conventional health education	Low risk	Cannot be ruled out	Present	Present	None (*P* = 0.837)	None (*P* = 0.837)	Low
TUGT	Offline traditional training vs. mHealth	Low risk	Cannot be ruled out	None	Present (k = 4)	None (*P* = 0.936)	None (*P* = 0.936)	Moderate
	Offline traditional training vs. Conventional home exercise	Low risk	Cannot be ruled out	Present	Present (k = 4)	None (*P* = 0.936)	None (*P* = 0.936)	Low
	Offline traditional training vs. Conventional in-person rehab	Low risk	Cannot be ruled out	Present	Present (k = 4)	None (*P* = 0.936)	None (*P* = 0.936)	Low
	Real-time Feedback vs. mHealth	Low risk	Cannot be ruled out	None	Present (k = 4)	None (*P* = 0.936)	None (*P* = 0.936)	Moderate
	Real-time Feedback vs. Traditional in-person training	Low risk	Cannot be ruled out	None	Present (k = 4)	None (*P* = 0.936)	None (*P* = 0.936)	Moderate
	mHealth vs. Conventional home exercise	Low risk	Cannot be ruled out	None	Present (k = 4)	None (*P* = 0.936)	None (*P* = 0.936)	Moderate

## Discussion

### Summary of findings

This systematic review and network meta-analysis included 8 RCTs to evaluate the effects of different digital health interventions on muscle health and physical function in older adults. The results showed that for muscle physiological adaptation outcomes, Real-time Feedback ranked highest in SUCRA for both SMI and handgrip strength, with league table results indicating statistically significant effects compared with conventional health education or habitual activities; however, these rankings are primarily based on indirect comparisons and should not be directly translated into definitive judgments of clinical superiority. For physical mobility function (TUGT), Offline traditional training ranked highest (SUCRA = 98.7%). Cluster analysis further confirmed the dual advantage of Real-time Feedback in the synergistic improvement of muscle strength and mass, while exploratory meta-regression suggested a positive distribution trend in intervention effects with increasing duration, particularly beyond 12 weeks. In addition, no significant publication bias or small-study effects were identified via comparison-adjusted funnel plots and Egger’s test (all P > 0.05), suggesting relatively high statistical robustness of the overall evidence network. CINeMA evidence certainty was overall rated as “low” to “moderate”; the efficacy ranking results of this study carry some evidence-based reference value, but given the limited number of included studies and the reliance of most comparisons on indirect evidence, related clinical recommendations should be interpreted with caution.

### Comparison with previous studies and mechanistic analysis

The findings of this study further extend those of Chen et al. (2025) [16] regarding digital interventions for sarcopenia. Their meta-analysis demonstrated that digital health interventions significantly improve skeletal muscle mass and handgrip strength, which is highly consistent with the trend observed in this study in which Real-time Feedback showed a relative advantage on the physiological adaptation indicators of handgrip strength and SMI. Notably, network comparisons in this study revealed that although digital interventions can improve muscle mass and strength, their effects on complex mobility functions such as TUGT remain substantially lower than those of Offline traditional training, suggesting that digital interventions may face certain challenges in translating muscle-level gains into complex neuromuscular coordination performance; however, given that only 4 studies reported TUGT, this conclusion requires further evidence. Furthermore, the findings of this study differ from those of Makizako et al. (2025) [14], whose study demonstrated significant functional improvement with digital interventions in healthy older adults, whereas participants with confirmed sarcopenia in the present study showed limited gains on TUGT. This discrepancy may be attributable to differences in functional baseline and degree of neuromuscular degeneration, suggesting that simple digital guidance may be insufficient to achieve rapid functional transfer in populations with severe functional impairment. On the other hand, this study aligns with Langeard et al. regarding the influence of technological interactivity. Langeard et al. noted uncertain evidence for wearable devices alone, while this study suggests through network meta-analysis that high-interactivity Real-time Feedback ranks relatively higher than low-interactivity modalities [15]. This consistency may be attributed to the capacity of high-interactivity technologies to provide real-time movement correction, addressing the core limitation of inadequate precise supervision in traditional digital interventions, thereby achieving superior physiological gains in muscle strength and mass.

The network synthesis results of this study showed that Real-time Feedback ranked highest among all digital intervention modalities for improving handgrip strength (SUCRA = 80.8%) and SMI (SUCRA = 78.3%). This relative advantage is hypothetically attributable to the immediate biofeedback mechanism it provides. Through AI-based correction or virtual reality interaction, this modality can offer precise movement trajectory guidance to older patients, helping participants maintain adequate mechanical load and correct biomechanical posture under digital supervision, thereby potentially facilitating physiological adaptive gains in skeletal muscle [34, 35]. In contrast, mHealth and Hardware-driven Monitoring modalities ranked lower on these indicators, hypothetically because they belong to asynchronous or low-interactivity categories and lack dynamic monitoring of movement quality, which may result in insufficient movement precision or subthreshold training intensity that fails to generate the expected physiological stimulus [36, 37]. In the comprehensive cluster analysis ranking, Real-time Feedback was identified as exhibiting a relative comprehensive advantage among digital modalities. This ranking result is hypothetically reflective of the synergistic effect of high-interactivity technology in simultaneously increasing skeletal muscle mass and improving muscle strength. Compared with single-dimension improvement, it is theoretically postulated that Real-time Feedback may achieve concurrent increases in muscle cross-sectional area and optimization of neural drive through sustained recruitment of the neuromuscular system [38, 39]. However, all of the above mechanistic hypotheses were not directly tested in the included trials and remain at the level of theoretical conjecture, pending verification by future research. For complex mobility outcomes such as TUGT, Offline traditional training achieved a high ranking (SUCRA = 98.7%). This indicates that although digital technologies have demonstrated favorable efficacy in improving physiological indicators such as muscle strength and mass, their capacity to translate these gains into complex neuromuscular coordination and dynamic balance output remains limited [40]. Within the evidence base of this study, screen-based or sensor-based digital approaches have not yet achieved effects on TUGT comparable to those of Offline traditional training; however, this does not imply that digital methods are ineffective for improving mobility. It more likely reflects the limitations of the currently included studies in terms of intervention content design and follow-up duration, and future purposefully designed digital balance and coordination training programs retain potential for improving mobility function [41]. Regarding clinical moderating factors, this study observed that intervention duration may influence outcomes. It is theoretically hypothesized that longer interventions (more than 12 weeks) tend to produce more stable muscle gains; however, given the limited number of relevant studies currently available, the evidence for this trend is insufficient and its reliability should be interpreted with caution. In addition, participants’ digital literacy is a key variable influencing intervention effects [42]. Insufficient age-friendly device design may lead to adherence bias, particularly in older patients who may experience frustration when operating complex devices, thereby diluting the overall intervention effect [43]. Furthermore, incomplete standardization of measurement protocols across included studies, such as variations in handgrip strength testing postures, while not causing statistical conflicts, should nonetheless be considered as a potential source of influence from experimental design heterogeneity when interpreting the relative ranking of interventions.

### Limitations

This study has the following limitations. First, the number of included RCTs was limited (*n* = 8), the overall network structure was sparse, and the vast majority of direct comparisons were supported by only a single study. Most rankings relied on indirect comparison inference, with wide confidence intervals; only 4 studies reported TUGT outcomes, resulting in particularly insufficient statistical power; and sensitivity analysis indicated that excluding high-risk-of-bias studies attenuated the statistical significance of some effect sizes, suggesting that current rankings are highly dependent on a small number of high-quality studies. These factors collectively result in considerable uncertainty in SUCRA rankings, which should be regarded as preliminary directional evidence rather than definitive conclusions, and should not serve as the sole basis for strong clinical recommendations.

Second, considerable heterogeneity existed across studies in terms of technological platforms, intervention durations (4 to 24 weeks), exercise intensity, and measurement protocols, increasing the difficulty of comparing intervention efficacy. Given that k ≤ 6 for each outcome in the exploratory meta-regression, statistical power was extremely limited, and dose-response trends are provided for hypothesis generation purposes only. Third, grey literature was not searched, and inclusion was restricted to Chinese and English publications, meaning that publication bias cannot be fully excluded. The number of included studies for each outcome was fewer than 10, limiting the statistical power of funnel plot inspection and Egger’s test; “no significant publication bias detected” does not confirm the absence of bias [44].

Fourth, all 8 studies were conducted in Asian populations. Systematic differences in participant body composition, diagnostic criteria, and digital infrastructure availability between Asian and Western populations limit the generalizability of the findings to non-Asian populations. Fifth, this study reflects intervention efficacy under idealized conditions rather than real-world effectiveness. The relatively high adherence rates observed in trials (83% to 96%) may not be replicable in routine clinical practice, as limited digital literacy among older adults, device operation barriers, and the absence of sustained professional supervision may all contribute to lower real-world effectiveness than trial results suggest. Follow-up durations were generally short (4 to 24 weeks), precluding evaluation of long-term adherence and sustained effects. Future research should adopt pragmatic RCT designs to evaluate the long-term effectiveness and sustainability of digital interventions across diverse cultural contexts.

### Practical implications

Within the current limited evidence base, digital platforms integrating Real-time Feedback technology demonstrated a relative advantage in improving SMI and handgrip strength in older adults with sarcopenia (CINeMA assessment overall at “moderate” certainty level), supported by a certain degree of evidence. Given the progressive nature of sarcopenia, even modest improvements in muscle mass and strength carry clinical significance in terms of reduced fall risk and preserved capacity for activities of daily living. In prescription development, prioritizing devices with AI movement recognition or biofeedback functions may help ensure training precision in remote settings, compensating for the absence of professional on-site supervision in traditional telerehabilitation. However, the above recommendations are based on SUCRA probability rankings; a higher ranking does not imply absolute superiority and should be applied with caution in the context of individual clinical circumstances.

Given the relatively high ranking of Offline traditional training for TUGT improvement, a hybrid management model combining “online real-time feedback with offline physical guidance” may be considered in clinical practice: offline guidance in the early intervention phase to ensure movement safety, followed by digital means during the maintenance phase for long-term functional consolidation. However, the wider implementation of a hybrid model faces multiple barriers, including high AI device costs, insufficient digital literacy among older patients, variation in technology accessibility across regions, and uncertain long-term adherence; clinical rollout therefore requires concurrent optimization of age-friendly design, tiered training support, and cost-effectiveness evaluation. Additionally, exploratory trends suggest that longer intervention durations (more than 12 weeks) may be associated with more robust effects, but given the current limited evidence base, this is insufficient to support standardized recommendations and warrants validation by future high-quality studies.

## Conclusion

This network meta-analysis of 8 RCTs found that digital health interventions show potential for improving muscle mass and strength in older adults with sarcopenia. Within the current available limited evidence, Real-time Feedback achieved the highest probability ranking for improving handgrip strength and SMI, suggesting that immediate interaction and movement correction may play a positive role in inducing neuromuscular adaptation, and showing some promise for clinical application. However, these conclusions are primarily based on indirect comparisons with a limited number of included studies and should not be interpreted as definitive evidence that Real-time Feedback is the optimal intervention. For improving complex mobility function, Offline traditional training ranked higher than digital interventions; however, given the low certainty of evidence for TUGT and the inclusion of only 4 studies, the clinical significance of this difference requires cautious interpretation. In summary, digital systems with high-interactivity features may serve as effective complementary strategies in the clinical management of sarcopenia, but their clinical value warrants confirmation by larger-scale, low-risk-of-bias head-to-head RCTs. Future research should also integrate physiological biomarkers to further elucidate the underlying mechanisms, and validate generalizability across diverse cultural contexts.

## Supplementary Information

Supplementary Material 1.

Supplementary Material 2.

Supplementary Material 3.

Supplementary Material 4.

## Data Availability

The datasets generated and/or analyzed during the current study are available from the corresponding author on reasonable request.
