# Supplementary material for: Effectiveness of different modes of digital health interventions for sarcopenia in older adults: a systematic review and network meta-analysis
Source: BMC Geriatr. 2026 Jul 21;26:1020. doi: 10.1186/s12877-026-07890-0 (PMC13449855; doi:10.1186/s12877-026-07890-0)
Supplement: Supplementary file 1 [file 12877_2026_7890_MOESM1_ESM.docx]

**Supplementary Text 1 Search Strategies**

**1.PubMed (February 13, 2026)**

**search strategy:**

| **Step** | **Search Terms and Boolean Logic** | **Results (n)** |
| --- | --- | --- |
| #1 | ("sarcopenia"[MeSH Terms]) | 14,653 |
| #2 | (Sarcopen*[Title/Abstract] OR "muscle wasting"[Title/Abstract] OR "muscle atrophy"[Title/Abstract] OR "muscle loss"[Title/Abstract]) | 45,964 |
| #3 | #1 OR #2 | 46,779 |
| #4 | ("Aged"[MeSH Terms]) | 3,849,710 |
| #5 | ("older people*"[Title/Abstract] OR "old age*"[Title/Abstract] OR adult*[Title/Abstract] OR "Aged, 60 and over"[Title/Abstract] OR "older age*"[Title/Abstract] OR "old people*"[Title/Abstract] OR geriatric*[Title/Abstract] OR "older adult*"[Title/Abstract] OR Senior*[Title/Abstract] OR Elderly[Title/Abstract]) | 2,366,447 |
| #6 | #4 OR #5 | 5,540,310 |
| #7 | ("Telemedicine"[MeSH Terms] OR "Virtual Reality"[MeSH Terms] OR "Artificial Intelligence"[MeSH Terms] OR "Wearable Electronic Devices"[MeSH Terms] OR "Video Games"[MeSH Terms] OR "Mobile Applications"[MeSH Terms] OR "Computers, Handheld"[MeSH Terms] OR "Reminder Systems"[MeSH Terms]) | 396,285 |
| #8 | ("telemedicine"[Title/Abstract] OR "telecommunication*"[Title/Abstract] OR "Telerehabilitation"[Title/Abstract] OR "telecare"[Title/Abstract] OR "Digital Technolog*"[Title/Abstract] OR "digital health"[Title/Abstract] OR "digital tool"[Title/Abstract] OR "digital care"[Title/Abstract] OR "mobile app*"[Title/Abstract] OR "mobileapp*"[Title/Abstract] OR "ehealth"[Title/Abstract] OR "e-health"[Title/Abstract] OR "mhealth"[Title/Abstract] OR "mobile health"[Title/Abstract] OR "online health"[Title/Abstract] OR "smartphone*"[Title/Abstract] OR "web-based"[Title/Abstract] OR "electronic monitoring"[Title/Abstract] OR "reminder device*"[Title/Abstract] OR "reminder system*"[Title/Abstract] OR "helping hand"[Title/Abstract] OR "Internet-Based Intervention"[Title/Abstract] OR "mobile phone*"[Title/Abstract] OR "cd-rom software"[Title/Abstract] OR "internet website*"[Title/Abstract] OR "e-mail contact*"[Title/Abstract] OR "sms based system*"[Title/Abstract] OR "telemedicine platform*"[Title/Abstract] OR "telehealth"[Title/Abstract] OR "eHealth intervention*"[Title/Abstract] OR "monitoring device*"[Title/Abstract] OR "mobile application*"[Title/Abstract] OR "digital assistant*"[Title/Abstract] OR "IoT"[Title/Abstract] OR "chat-bot"[Title/Abstract] OR "chatbot"[Title/Abstract] OR "health website*"[Title/Abstract] OR "metaverse"[Title/Abstract] OR "Exergaming"[Title/Abstract] OR "esports"[Title/Abstract] OR "e-sports"[Title/Abstract] OR "wearable"[Title/Abstract] OR "smartwatch"[Title/Abstract] OR "smart watch"[Title/Abstract] OR "digital*"[Title/Abstract] OR "activity tracker*"[Title/Abstract] OR "virtual*"[Title/Abstract] OR "Mixed Reality"[Title/Abstract] OR "Augmented Reality"[Title/Abstract] OR "exergame*"[Title/Abstract] OR "video conferenc*"[Title/Abstract] OR "Machine Learning"[Title/Abstract] OR "Deep Learning"[Title/Abstract] OR "remote intervention*"[Title/Abstract] OR "Nintendo Switch"[Title/Abstract] OR "Kinect"[Title/Abstract]) | 934,786 |
| #9 | #7 OR #8 | 1,116,459 |
| #10 | ("randomized controlled trial"[Publication Type]) | 657,842 |
| #11 | ("randomized"[Title/Abstract] OR "placebo"[Title/Abstract]) | 947,089 |
| #12 | #10 OR #11 | 1,183,552 |
| #13 | #3 AND #6 AND #9 AND #12 | 472 |

**2.Web of Science (February 13, 2026)**

**search strategy:**

| **Step** | **Search Strategy (TS)** | **Results (n)** |
| --- | --- | --- |
| #1 | (sarcopenia OR "muscle wasting" OR "muscle atrophy" OR "muscle loss") | 113897 |
| #2 | ("older people" OR adult OR "old age" OR "Aged, 60 and over" OR Aged OR "older age" OR "old people" OR geriatric OR "older adult" OR Senior OR Elderly) | 5235992 |
| #3 | (telemedicine OR telecommunication OR Telerehabilitation OR telecare OR "Digital Technolog" OR "digital health" OR "digital tool" OR "digital care" OR "mobile app" OR mobileapp OR ehealth OR e-health OR mhealth OR "mobile health" OR "online health" OR "Computers, Handheld" OR "Reminder Systems" OR smartphone OR web-based OR "electronic monitoring" OR "reminder device" OR "reminder system" OR "helping hand" OR "Internet-Based Intervention" OR "mobile phone" OR "cd-rom software" OR "internet website" OR "e-mail contact" OR "sms based system" OR "telemedicine platform" OR telehealth OR "eHealth intervention" OR "monitoring device" OR "mobile application" OR "digital assistant" OR "Artificial Intelligence" OR IoT OR chat-bot OR chatbot OR "health website" OR "Virtual Reality" OR metaverse OR Exergaming OR esports OR e-sports OR "Wearable Electronic Devices" OR wearable OR smartwatch OR "smart watch") | 871292 |
| #4 | ("randomized controlled trial" OR randomized OR placebo) | 1195281 |
| #5 | #1 AND #2 AND #3 AND #4 | 860 |

**3.Cochrane Library (February 13, 2026)**

**search strategy:**

| **Step** | **Search Strategy (ti, ab, kw)** | **Results (n)** |
| --- | --- | --- |
| #1 | (sarcopenia OR "muscle wasting" OR "muscle atrophy" OR "muscle loss") | 15,039 |
| #2 | ("older people" OR adult OR "old age" OR "Aged, 60 and over" OR Aged OR "older age" OR "old people" OR geriatric OR "older adult" OR Senior OR Elderly) | 1,222,023 |
| #3 | (telemedicine OR telecommunication OR Telerehabilitation OR telecare OR "Digital Technolog" OR "digital health" OR "digital tool" OR "digital care" OR "mobile app" OR mobileapp OR ehealth OR e-health OR mhealth OR "mobile health" OR "online health" OR "Computers, Handheld" OR "Reminder Systems" OR smartphone OR web-based OR "electronic monitoring" OR "reminder device" OR "reminder system" OR "helping hand" OR "Internet-Based Intervention" OR "mobile phone" OR "cd-rom software" OR "internet website" OR "e-mail contact" OR "sms based system" OR "telemedicine platform" OR telehealth OR "eHealth intervention" OR "monitoring device" OR "mobile application" OR "digital assistant" OR "Artificial Intelligence" OR IoT OR chat-bot OR chatbot OR "health website" OR "Virtual Reality" OR metaverse OR Exergaming OR esports OR e-sports OR "Wearable Electronic Devices" OR wearable OR smartwatch OR "smart watch") | 109,182 |
| #4 | ("randomized controlled trial" OR randomized OR placebo) | 1,514,982 |
| #5 | #1 AND #2 AND #3 AND #4 | 1,842 |

**4.Scopus (February 13, 2026)**

**search strategy:**

| **Step** | **Search Strategy (TITLE-ABS-KEY)** | **Results (n)** |
| --- | --- | --- |
| #1 | (sarcopenia OR "muscle wasting" OR "muscle atrophy" OR "muscle loss") | 123,421 |
| #2 | ("older people" OR adult OR "old age" OR "Aged, 60 and over" OR Aged OR "older age" OR "old people" OR geriatric OR "older adult" OR Senior OR Elderly) | 58,115 |
| #3 | (telemedicine OR telecommunication OR Telerehabilitation OR telecare OR "Digital Technolog" OR "digital health" OR "digital tool" OR "digital care" OR "mobile app" OR mobileapp OR ehealth OR e-health OR mhealth OR "mobile health" OR "online health" OR "Computers, Handheld" OR "Reminder Systems" OR smartphone OR web-based OR "electronic monitoring" OR "reminder device" OR "reminder system" OR "helping hand" OR "Internet-Based Intervention" OR "mobile phone" OR "cd-rom software" OR "internet website" OR "e-mail contact" OR "sms based system" OR "telemedicine platform" OR telehealth OR "eHealth intervention" OR "monitoring device" OR "mobile application" OR "digital assistant" OR "Artificial Intelligence" OR IoT OR chat-bot OR chatbot OR "health website" OR "Virtual Reality" OR metaverse OR Exergaming OR esports OR e-sports OR "Wearable Electronic Devices" OR wearable OR smartwatch OR "smart watch") | 2,474,210 |
| #4 | ("randomized controlled trial" OR randomized OR placebo) | 1,287,035 |
| #5 | #1 AND #2 AND #3 AND #4 | 1,889 |

**5.Embase (February 13, 2026)**

**search strategy:**

| **Step** | **Search Strategy (ti, ab, kw)** | **Results (n)** |
| --- | --- | --- |
| #1 | (sarcopenia OR "muscle wasting" OR "muscle atrophy" OR "muscle loss") | 68,043 |
| #2 | ("older people" OR adult OR "old age" OR "Aged, 60 and over" OR Aged OR "older age" OR "old people" OR geriatric OR "older adult" OR Senior OR Elderly) | 2,074,320 |
| #3 | (telemedicine OR telecommunication OR Telerehabilitation OR telecare OR "Digital Technolog" OR "digital health" OR "digital tool" OR "digital care" OR "mobile app" OR mobileapp OR ehealth OR e-health OR mhealth OR "mobile health" OR "online health" OR "Computers, Handheld" OR "Reminder Systems" OR smartphone OR web-based OR "electronic monitoring" OR "reminder device" OR "reminder system" OR "helping hand" OR "Internet-Based Intervention" OR "mobile phone" OR "cd-rom software" OR "internet website" OR "e-mail contact" OR "sms based system" OR "telemedicine platform" OR telehealth OR "eHealth intervention" OR "monitoring device" OR "mobile application" OR "digital assistant" OR "Artificial Intelligence" OR IoT OR chat-bot OR chatbot OR "health website" OR "Virtual Reality" OR metaverse OR Exergaming OR esports OR e-sports OR "Wearable Electronic Devices" OR wearable OR smartwatch OR "smart watch") | 457,078 |
| #4 | ("randomized controlled trial" OR randomized OR placebo) | 1,582,605 |
| #5 | #1 AND #2 AND #3 AND #4 | 1,287 |

**6.CINAHL (February 13, 2026)**

**search strategy:**

| **Step** | **Search Strategy (XB)** | **Results (n)** |
| --- | --- | --- |
| #1 | (sarcopenia OR "muscle wasting" OR "muscle atrophy" OR "muscle loss") | 14,217 |
| #2 | ("older people" OR adult OR "old age" OR "Aged, 60 and over" OR Aged OR "older age" OR "old people" OR geriatric OR "older adult" OR Senior OR Elderly) | 2,380,763 |
| #3 | (telemedicine OR telecommunication OR Telerehabilitation OR telecare OR "Digital Technolog" OR "digital health" OR "digital tool" OR "digital care" OR "mobile app" OR mobileapp OR ehealth OR e-health OR mhealth OR "mobile health" OR "online health" OR "Computers, Handheld" OR "Reminder Systems" OR smartphone OR web-based OR "electronic monitoring" OR "reminder device" OR "reminder system" OR "helping hand" OR "Internet-Based Intervention" OR "mobile phone" OR "cd-rom software" OR "internet website" OR "e-mail contact" OR "sms based system" OR "telemedicine platform" OR telehealth OR "eHealth intervention" OR "monitoring device" OR "mobile application" OR "digital assistant" OR "Artificial Intelligence" OR IoT OR chat-bot OR chatbot OR "health website" OR "Virtual Reality" OR metaverse OR Exergaming OR esports OR e-sports OR "Wearable Electronic Devices" OR wearable OR smartwatch OR "smart watch") | 201,234 |
| #4 | ("randomized controlled trial" OR randomized OR placebo) | 416,889 |
| #5 | #1 AND #2 AND #3 AND #4 | 368 |

**7.CNKI (February 13, 2026)**

**search strategy:**

| **Step** | **Search Strategy (Subject/Keyword)** | **Results (n)** |
| --- | --- | --- |
| #1 | (老年人 OR 老年人口 OR 老年人群 OR "60岁及以上老年人") | 233,888 |
| #2 | (肌少症 OR "肌少症患者") | 2,865 |
| #3 | (干预 OR 干预前后 OR 干预研究 OR 干预效果) | 591,153 |
| #4 | #1 AND #2 AND #3 | 137 |
