# Supplementary material for: Effectiveness of different modes of digital health interventions for sarcopenia in older adults: a systematic review and network meta-analysis
Source: BMC Geriatr. 2026 Jul 21;26:1020. doi: 10.1186/s12877-026-07890-0 (PMC13449855; doi:10.1186/s12877-026-07890-0)
Supplement: Supplementary file 2 [file 12877_2026_7890_MOESM2_ESM.docx]

Supplementary Table 1 Summary of Global Consistency Testing and Model Fit Results for Primary Outcomes in the Network Meta-Analysis

| **Outcomes** | **Number of Comparisons (m)** | **Number of Interventions (n)** | **Global Inconsistency Q Statistic** | **Degrees of Freedom (df)** | **P-value (Global)** |
| --- | --- | --- | --- | --- | --- |
| Handgrip Strength | 10 | 8 | 0.058 | 1 | 0.81 |
| Skeletal Muscle Index (SMI) | 11 | 9 | 0.042 | 1 | 0.837 |
| Timed Up and Go Test (TUGT) | 8 | 6 | 0.006 | 1 | 0.936 |

Supplementary Table 2 Summary of Consistency Testing for Primary Outcomes Using the Node-Splitting Method in Closed Loops

| **Outcome** | **Comparison** | **Direct** | **Indirect** | **P-consistency** |
| --- | --- | --- | --- | --- |
| Handgrip | mHealth vs. Offline traditional training | 0.053 | -0.137 | 0.81 |
|  | Real-time Feedback vs. Traditional in-person | 0.036 | -0.162 | 0.81 |
| SMI | mHealth vs. Offline traditional training | 0.099 | -0.071 | 0.837 |
|  | Real-time Feedback vs. Traditional in-person | 0.064 | -0.106 | 0.837 |
| TUGT | mHealth vs. Offline traditional training | 0.938 | 0.869 | 0.936 |
|  | Real-time Feedback vs. Traditional in-person | 0.009 | -0.057 | 0.936 |

Supplementary Table 3 Summary of SUCRA Values and Rankings for Handgrip Strength

| **Rank** | **Intervention** | **P-score (SUCRA)** |
| --- | --- | --- |
| 1 | Real-time Feedback | 0.8077 |
| 2 | Traditional in-person training | 0.7396 |
| 3 | Conventional in-person rehab | 0.6996 |
| 4 | mHealth | 0.5968 |
| 5 | Offline traditional training | 0.5087 |
| 6 | Conventional home exercise | 0.4499 |
| 7 | Hardware-driven Monitoring | 0.4258 |
| 8 | Conventional health education | 0.2647 |
| 9 | Habitual activities | 0.0072 |

Supplementary Table 4 Summary of SUCRA Values and Rankings for Skeletal Muscle Mass Index

| **Rank** | **Intervention** | **P-score (SUCRA)** |
| --- | --- | --- |
| 1 | Real-time Feedback | 0.7829 |
| 2 | Traditional in-person training | 0.7562 |
| 3 | mHealth | 0.7129 |
| 4 | Offline traditional training | 0.6784 |
| 5 | Conventional in-person rehab | 0.5901 |
| 6 | Hardware-driven Monitoring | 0.2912 |
| 7 | Conventional health education | 0.1567 |
| 8 | Habitual activities | 0.0316 |

Supplementary Table 5 Summary of SUCRA Values and Rankings for the Timed Up and Go Test (TUGT)

| **Rank** | **Intervention** | **P-score (SUCRA)** |
| --- | --- | --- |
| 1 | Offline traditional training | 0.9867 |
| 2 | Real-time Feedback | 0.5126 |
| 3 | Traditional in-person training | 0.498 |
| 4 | Conventional home exercise | 0.3611 |
| 5 | Conventional in-person rehab | 0.3594 |
| 6 | mHealth | 0.2823 |

Supplementary Table 6 League Table of the Network Meta-Analysis for the Effects of Digital Health Interventions on Handgrip Strength in Older Adults

| Handgrip Strength | 1. Conventional health education | 2. Conventional in-person rehab | 3. Habitual activities | 4. Hardware-driven Monitoring | 5. mHealth | 6. Offline traditional training | 7. Real-time Feedback | 8. Traditional in-person training |
| --- | --- | --- | --- | --- | --- | --- | --- | --- |
| 1. Conventional health education | Conventional health education |  |  |  |  |  |  |  |
| 2. Conventional in-person rehab | 0.72 (0.07 to 1.36) | Conventional in-person rehab |  |  |  |  |  |  |
| 3. Habitual activities | -0.34 (-1.07 to 0.38) | -1.06 (-2.03 to -0.09) | Habitual activities |  |  |  |  |  |
| 4. Hardware-driven Monitoring | 0.20 (-0.38 to 0.77) | -0.52 (-1.38 to 0.34) | 0.54 (0.09 to 0.98) | Hardware-driven Monitoring |  |  |  |  |
| 5. mHealth | 0.85 (0.51 to 1.20) | 0.14 (-0.41 to 0.69) | 1.20 (0.40 to 2.00) | 0.66 (-0.01 to 1.33) | mHealth |  |  |  |
| 6. Offline traditional training | 0.83 (0.20 to 1.45) | 0.11 (-0.65 to 0.87) | 1.17 (0.21 to 2.13) | 0.63 (-0.21 to 1.48) | -0.03 (-0.49 to 0.55) | Offline traditional training |  |  |
| 7. Real-time Feedback | 0.92 (0.40 to 1.44) | 0.21 (-0.47 to 0.88) | 1.26 (0.37 to 2.16) | 0.73 (-0.05 to 1.50) | 0.07 (-0.33 to 0.46) | 0.10 (-0.42 to 0.61) | Real-time Feedback |  |
| 8. Traditional in-person training | 0.91 (0.28 to 1.55) | 0.20 (-0.57 to 0.96) | 1.25 (0.29 to 2.22) | 0.72 (-0.14 to 1.57) | 0.06 (-0.48 to 0.59) | 0.08 (-0.61 to 0.77) | -0.01 (-0.52 to 0.55) | Traditional in-person training |

Supplementary Table 7 League Table of the Network Meta-Analysis for the Effects of Digital Health Interventions on Skeletal Muscle Mass in Older Adults

| Skeletal Muscle Mass | 1. Conventional health education | 2. Conventional home exercise | 3. Conventional in-person rehab | 4. Habitual activities | 5. Hardware-driven Monitoring | 6. mHealth | 7. Offline traditional training | 8. Real-time Feedback | 9. Traditional in-person training |
| --- | --- | --- | --- | --- | --- | --- | --- | --- | --- |
| 1. Conventional health education | Conventional health education |  |  |  |  |  |  |  |  |
| 2. Conventional home exercise | 0.15 (-0.74 to 1.03) | Conventional home exercise |  |  |  |  |  |  |  |
| 3. Conventional in-person rehab | 0.44 (-0.20 to 1.08) | 0.29 (-0.69 to 1.28) | Conventional in-person rehab |  |  |  |  |  |  |
| 4. Habitual activities | -0.92 (-1.66 to -0.18) | -1.07 (-2.22 to 0.08) | -1.36 (-2.34 to -0.38) | Habitual activities |  |  |  |  |  |
| 5. Hardware-driven Monitoring | 0.13 (-0.45 to 0.70) | -0.02 (-1.07 to 1.03) | -0.31 (-1.17 to 0.54) | 1.05 (0.58 to 1.51) | Hardware-driven Monitoring |  |  |  |  |
| 6. mHealth | 0.33 (0.00 to 0.66) | 0.18 (-0.64 to 1.00) | -0.11 (-0.66 to 0.44) | 1.25 (0.44 to 2.06) | 0.20 (-0.46 to 0.86) | mHealth |  |  |  |
| 7. Offline traditional training | 0.25 (-0.37 to 0.87) | 0.10 (-0.87 to 1.08) | -0.19 (-0.95 to 0.57) | 1.17 (0.21 to 2.13) | 0.12 (-0.72 to 0.97) | -0.08 (-0.60 to 0.45) | Offline traditional training |  |  |
| 8. Real-time Feedback | 0.53 (0.01 to 1.04) | 0.38 (-0.53 to 1.29) | 0.08 (-0.59 to 0.76) | 1.45 (0.55 to 2.35) | 0.40 (-0.37 to 1.17) | 0.20 (-0.20 to 0.59) | 0.28 (-0.26 to 0.82) | Real-time Feedback |  |
| 9. Traditional in-person training | 0.48 (-0.15 to 1.11) | 0.34 (-0.64 to 1.32) | 0.04 (-0.73 to 0.81) | 1.40 (0.43 to 2.38) | 0.36 (-0.49 to 1.21) | 0.16 (-0.38 to 0.69) | 0.23 (-0.41 to 0.87) | -0.05 (-0.59 to 0.49) | Traditional in-person training |

Supplementary Table 8 League Table of the Network Meta-Analysis for the Effects of Digital Health Interventions on the Timed Up and Go Test (TUGT) in Older Adults

| Timed Up and Go Test | 1. Conventional home exercise | 2. Conventional in-person rehab | 3. mHealth | 4. Offline traditional training | 5. Real-time Feedback | 6. Traditional in-person training |
| --- | --- | --- | --- | --- | --- | --- |
| 1. Conventional home exercise | Conventional home exercise |  |  |  |  |  |
| 2. Conventional in-person rehab | -0.01 (-1.00 to 0.97) | Conventional in-person rehab |  |  |  |  |
| 3. mHealth | 0.02 (-0.80 to 0.84) | 0.04 (-0.51 to 0.58) | mHealth |  |  |  |
| 4. Offline traditional training | -0.91 (-1.89 to 0.07) | -0.89 (-1.67 to -0.12) | -0.93 (-1.47 to -0.39) | Offline traditional training |  |  |
| 5. Real-time Feedback | -0.14 (-1.05 to 0.76) | -0.13 (-0.81 to 0.55) | -0.16 (-0.56 to 0.23) | 0.77 (0.23 to 1.30) | Real-time Feedback |  |
| 6. Traditional in-person training | -0.15 (-1.12 to 0.83) | -0.13 (-0.90 to 0.64) | -0.17 (-0.70 to 0.37) | 0.76 (0.06 to 1.47) | 0.00 (-0.54 to 0.53) | Traditional in-person training |

Supplementary Figure 1 SUCRA cumulative ranking probability plots for different interventions

Note: (A) Handgrip Strength: Representing the potential of interventions to improve muscle strength； (B) Skeletal Muscle Index (SMI): Representing the potential to increase muscle mass； (C) Timed Up and Go Test (TUGT): Representing the improvement in physical functional mobility.
